# Mixtures of fluopyram and abamectin for management of *Meloidogyne incognita* in tomato

**DOI:** 10.21307/jofnem-2020-129

**Published:** 2021-01-13

**Authors:** Qing-Qing Li, Jing-Jing Li, Qi-Tong Yu, Ze-Yu Shang, Chao-Bin Xue

**Affiliations:** Key Laboratory of Pesticide Toxicology and Application Technique, College of Plant Protection, Shandong Agricultural University, Tai’an, China

**Keywords:** Abamectin, Control efficacy, Fluopyram, Root-galling index, Root-knot nematode

## Abstract

The southern root-knot nematode (RKN), *Meloidogyne incognita*, causes significant damage to vegetable production and is a major problem in greenhouse tomatoes. The effect of a combination of fluopyram and abamectin, at a mass ratio of 1:5, was studied for RKN control. Pot trials showed that fluopyram, abamectin, and their combination at three dosages increased the height, stem diameter, root fresh weight, shoot fresh weight, and the root length of tomato plants. The RKN control efficacy of the 1:5 combination at 450 g a.i./ha was 74.06% at 30 days after transplanting (DAT), and the control efficacy of the combination at 337.5 and 450 g a.i./ha differed significantly from those of other treatments at 60 DAT. The root-galling index (RGI) control efficacy of the combination at 450 g a.i./ha and of fluopyram (41.7% SC) only at 450 g a.i./ha were better than the control efficacies of other treatments, and these two treatments significantly increased root activity. Field trial results showed that the soil nematode control efficacy was similar to that of the pot trials at 30 and 60 DAT. The RGI control efficacy of the combination at 337.5 and 450 g a.i./ha and of fluopyram (41.7% SC) only at 450 g a.i./ha differed significantly from those of the two other treatments. The tomato yields of the 1:5 combination at 450 g a.i./ha were increased by 24.07 and 23.22% compared to the control in field trials during two successive years. The combination of fluopyram and abamectin provides good nematode measure, and it can increase tomato yields. It provides an effective solution for the integrated management of southern RKN.

Nematodes are important parasites of crops. The economic losses caused by nematodes worldwide exceed 157 billion US dollars annually (Abad et al., 2008). Root-knot nematodes have a wide host range and are especially harmful to plants in the Cucurbitaceae and Solanaceae (Nicol et al., 2011). Tomato is extensively cultivated worldwide and highly susceptible. When the southern root-knot nematode (RKN) *Meloidogyne incognita* infects tomato, the second-stage juveniles (J2) penetrate young roots, causing root galls that disrupt water relations and the physiology of infected plants. This can result in severe stunting of the plant (Pinkerton and Finn, 2005). The RKN can also interact with other pathogenic microorganisms, causing serious diseases in tomato (Tian et al., 2015). The damage caused by the RKN to tomato reduces production by 10–30% and losses can exceed 50% in northern China (Sun et al., 2006; Huang et al., 2019a).

Nematodes have historically been controlled by applications of soil fumigants. However, the use of fumigants such as methyl bromide and 1,3-dichloropropene is now banned or restricted (Giannakou et al., 2005). Some non-fumigant nematicides, such as aldicarb and carbofuran, are restricted because of their high mammalian toxicity and environmental risks (Shi et al., 2019). Although current agricultural, physical, and biological control measures are used alone or in combination, the RKN is not effectively controlled in China.

Fluopyram, a systemic pesticide used for the control of fungal diseases, is being evaluated for its nematicidal activity (Faske and Hurd, 2015). Fluopyram inhibits succinate dehydrogenase (SDH) in the tricarboxylic acid cycle and blocks electron transport in the mitochondria of fungi (Abad-Feuntes et al., 2015; Huang et al., 2019b). It has low toxicity to mammals, is environmentally friendly, and has been proposed as a nematicide for use in vegetable production (Jones et al., 2017). The application of fluopyram to soil has shown potential for the control of *Belonolaimus longicaudatus* nematode populations in Florida (USA) strawberries (Watson and Desaeger, 2019; Watson et al., 2020). The nematicide Velum^@^, with fluopyram as its main active ingredient, successfully controlled *M. incognita* populations in tomato (Dahlin et al., 2019).

Avermectins are macrocyclic lactones derived from the fermentation of *Streptomyces avermitilis* (Putter et al., 1981). Abamectin, with avermectins B1a and B1b as the active ingredients, affects the *γ*-amino butyric acid (GABA) nervous system of nematodes. It has been widely used for nematode control because of its nematicidal properties (Qiao et al., 2012; Shaver et al., 2016; Zhang et al., 2017). Abamectin can be used in combination with other nematicides, such as 1,3-dichloropropene (1,3-D), fosthiazate, and microbial nematicides. These combinations have potential for controlling *M. incognita* in tomato and cucumber fields in China (Qiao et al., 2012; Huang et al., 2016; Qiao et al., 2014; Huang et al., 2014). Abamectin can reduce populations of *Meloidogyne paranaensis* in coffee fields during transplantation, or 60 days later, and it is not phytotoxic to coffee plants (Arita et al., 2020). The application of abamectin has been an important component of an integrated pest management strategy for nematodes.

In this study, we combined fluopyram and abamectin to evaluate their management potential for *M. incognita* in infested tomatoes. We evaluated growth rate indicators, the root-galling index (RGI), number of nematodes in soil, and tomato yield. Our objective was to evaluate an effective mixture option for the integrated management of RKN.

## Materials and methods

### Preparation of *M. incognita*

Tomato roots with numerous *M. incognita* root knots were collected from a solar greenhouse in Daiyue (DY), Tai’an City, Shandong Province, China, and cut into smaller sections. The egg masses were collected from the knots using a dissecting needle under a microscope, soaked in a 0.2% NaOCl solution for 30 sec, and then washed several times with sterile water (Hussey and Barker, 1973). The eggs were then incubated for 2 or 3 days in an incubator at 25°C and sterile water was changed daily. The hatched second-stage juveniles (J2) in suspension were collected. The J2 nematode suspension was stored at 25°C and tested within two days.

### Chemicals

Pure fluopyram (97.5%, Sigma-Aldrich, Inc., St. Louis, MO, USA) and abamectin (94.8%, Qingdao Dongsheng Pharmaceutical Co., Ltd) and commercial pesticides based on fluopyram (41.7% SC, Bayer Crop Science Company, China), abamectin (1.8% EC, Henan Xinnong Chemical Co., Ltd), and triphenyltetrazolium chloride (TTC, 98%, Shanghai Fortuneibo Tech Co., Ltd) were used in this study. All other reagents used were analytically pure.

### Toxicity in vitro

Fluopyram (97.5%) or abamectin (94.8%) was dissolved in acetone, to obtain 2,000 μg/mL solutions, which were diluted using a 0.1% Tween 80 aqueous solution. The dilution concentrations were 0.10, 0.30, 0.90, 2.70, 8.10 μg/mL and 0.30, 0.60, 1.20, 2.40, 4.80 μg/mL for fluopyram and abamectin, respectively. Then, 500 μL of each dilution was added to a J2 nematode suspension of 500 μL (about 120 nematodes) in each well of the 12-well plates. The plates were sealed with food-grade Saran wrap to prevent volatilization and were placed in an incubator at 25 ± 2°C. All treatments were observed under a microscope after 48 h. Then, contact with the treated nematodes was made very slightly using a fine needle and the nematodes were identified as dead if they did not move. The total number of nematodes and the number of living nematodes were recorded, and the LC_50_ (i.e., lethal concentration required to kill 50% of the population) values were calculated by PROBIT analysis using SPSS software. The untreated control (Ctrl) consisted of equivalent amount of acetone in a 0.1% Tween 80 aqueous solution. The toxicity experiment was independently replicated three times.

Different combinations of 97.5% fluopyram and 94.8% abamectin (mass ratios of 1:1, 1:3, 1:5, 3:1, and 5:1) were also used to determine in vitro toxicity using the laboratory method mentioned above. The final concentration of the active ingredients of each combination was kept the same as that for the individual pesticides alone. The co-toxicity coefficient (CTC) of each combination was calculated according to Sun and Johnson (1960), and the most effective combination of commercial pesticides fluopyram (41.7% SC) and abamectin (1.8% EC) at an active ingredient (a.i.) mass ratio of 1:5 was used in the pot and field trials.

### Pot trials

**Figure 1:**
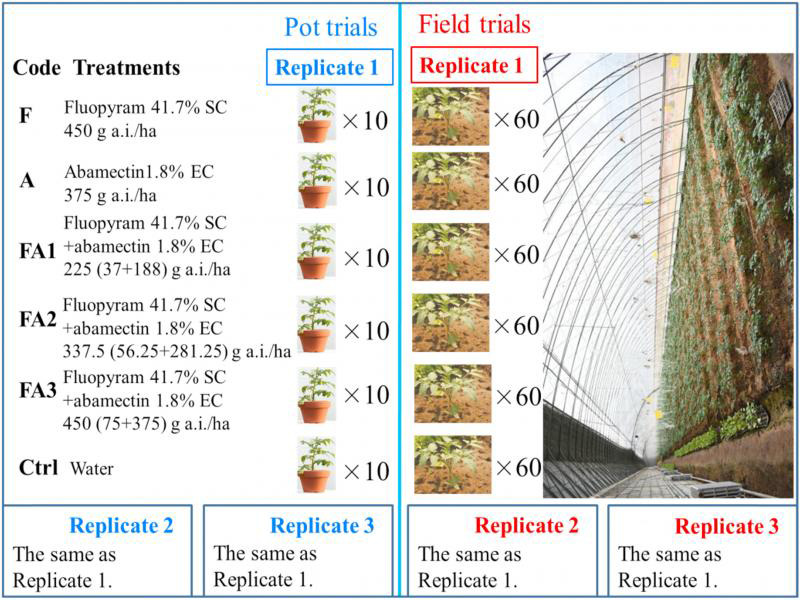
Experiments design of the pot and field trials.

Pot trials were conducted in a greenhouse of Shandong Agricultural University, Tai’an, China. The soil used in the clay pots (d × h, 24 × 26.5 cm) was a commercial vermiculite and silt loam without any nematodes. The soil had an organic matter content of 16.2 g/kg soil, pH of 6.9 (1: 5 soil: water), and available N, P, and K of 62.1 mg/kg, 7.8 mg/kg, and 221.5 mg/kg, respectively. Pots were filled with 2 kg of soil that was moistened before transplantation. One-month-old tomato seedlings (cv. JinPeng 11-8, four to five true leaf stage, susceptible to *M. incognita*) were transplanted into the pots (one seedling per pot). Each seedling was inoculated with about 1800 freshly hatched J2 of *M. incognita* in a 20 mL suspension, by injecting the nematode suspension into four 5 cm deep holes in the plant root rhizosphere soil. Two days after inoculation, 250 mL of different water-diluted commercial pesticide solutions were used for the soil drench. Water alone was used for the Ctrl treatment (Figure 1). Chemical application rates were based on the label application directions (ICAMA, 2019). The tomato plants received natural light about 10 h, with day and night average temperatures of approximately 30 and 20°C, respectively, and 60–70% relative humidity. Each treatment included 10 pots, and all treatments were independently replicated three times.

### Growth rate of indicator

The stem diameter and plant height of the tomatoes were measured at 30 and 60 days after transplanting (DAT). The stem diameter was measured with Vernier callipers. The root length and the fresh weights of the root and shoot were measured at 60 DAT with a ruler and a balance, respectively. The relative growth rate of each physiological growth indicator (*I*) was calculated to evaluate the growth status of tomato plants as follows:

GrowthrateofI(%)=100%×(treatedI−CtrlI)/CtrlI.

### Root activity

The root activity reflects the ability of root system to absorb water and nutrients directly, which is an indicator of plant health. The root activity of tomato was determined according to the TTC (triphenyltetrazolium chloride) method (Li, 2000). First, a TTC standard curve was obtained using the following method. A small amount (0.2 mL) of 0.4% TTC solution and 9.8 mL of methanol solution were placed in a 20 mL centrifuge tube; 0.10 g of Na_2_S_2_O_4_ was added. The solution was shaken well, and an 80 μg/mL concentration of red TTF (triphenylformazan) was produced. Then, 0.25 mL, 0.50 mL, 1.00 mL, 1.50 mL, and 2.00 mL of the red TTF solution were diluted to 10 mL with methanol to obtain 2 μg/mL, 4 μg/mL, 8 μg/mL, 12 μg/mL, and 16 μg/mL TTF standard solutions, respectively. The absorbance of each TTF standard solution was measured at 485 nm with a spectrophotometer (Epoch 2, Biotek, Winooski, VT, USA). A methanol solution was used as the control. The TTC standard curve was then calculated.

Root activity was determined as follows. Tomato root tips (0.5 cm in length, 0.20 g in total) of each treatment was placed in a culture dish, and 5 mL of 0.4% TTC solution and 5 mL of 0.1 mol/L PBS buffer (pH = 7.5) were added. The culture dish was placed in a 37°C incubator for 2.5 h for a shading reaction, and then a 2 mL solution of 1 mol/L H_2_SO_4_ was added. The Ctrl group received a 2 mL solution of 1 mol/L H_2_SO_4_ in the culture dish, followed by the addition of 0.2 g of root tip samples. The culture dish was placed in a 37°C incubator for 2.5 h for a shading reaction. The stained root samples were then removed and placed in a centrifuge tube with 10 mL methanol for decolorization. The centrifuge tube was placed in a 30°C incubator for 5 h. The absorbance of each methanol extract was measured at 485 nm with a spectrophotometer (Epoch 2, Biotek, Winooski, VT, USA). The content of TTF (mTTF) was calculated from the standard curve. The root activity was obtained according to the following formula:

Rootactivity(μg/g·h)=mTTF/(freshrootweight×reactiontime).

### Soil nematode and RGI control efficacy

Soil was collected from each pot of the treatment. Plant debris was removed, and soil was filtered with a 500-mesh sieve (25 μm) and mixed to get 100 g of soil samples for each pot independently. Nematodes were extracted from soil samples using the shallow dish method (Mao et al., 2004). The mortality and control efficacy of nematodes in soil were calculated using the following formulas:

Mortality(%)=(No.ofnematodesuntreated−No.ofnematodestreated)/No.ofnematodesuntreated×100%,

Controlefficacy(%)=(mortalityofCtrl−mortalityoftreated)/mortalityofCtrl×100%.

The RGI was determined by digging up all the roots of tomato plants in each treatment and evaluating root damage according to a scale of 0–10, where 0–10 represented no galls, 0–10%, 10–20%, 20–30%, 30–40%, 40–50%, 50–60%, 60–70%, 70–80%, 80–90%, and 90–100% galled roots, respectively (Barker et al., 1986). The RGI and control efficacy were calculated using the following formulas:

RGI=[Σ(No.ofplantsatallscales×itsscale)/(totalNo.ofplants×thehighestscale)]×100,

Controlefficacy=(RGIofCtrl−RGIoftreated)/RGIofCtrl×100%.

### Field trials

The field trials were conducted in a solar greenhouse in Laiwu (LW), Jinan City in March 2018, and in a solar greenhouse in Daiyue (DY), Tai’an City in November 2019, Shandong Province, with loam and sandy loam soil, respectively. Southern RKN have been a serious problem for tomatoes in both the fields for more than 10 years. The initial number of southern RKN in the field soil ranged from 1200 to 1800 per 100 cc (g) soil. We arranged six treatments randomly allocated to each of three blocks in the test field. The area of each plot was 24 m^2^ and it contained 60 tomato (JinPeng 11-8) plants. The tomato roots were irrigated by hand with pesticide solutions at 2 DAT; tomato seedlings were irrigated with 400 mL of a solution of the respective pesticide dilutions, while water was used for the Ctrl treatment. The dosages of the active ingredient of the pesticides (Figure 1), the calculation methods for the nematode control efficacy in the field rhizosphere soil, and RGI were the same as those used in the pot trials.

To assess tomato yield, the number of tomatoes was counted on 10 randomly selected tomato plants per plot after 12 and 15 weeks of treatment (Lu et al., 2017). A total of 20 randomly selected tomato fruits were weighed and their mean weight was calculated. Yield was measured twice (Qiao et al., 2011). The field trial was repeated twice (March 2018 and November 2019).

### Statistical analysis

We analyzed data using analysis of variance (ANOVA) and evaluated differences among the means by Tukey’s multiple comparison test (*p* = 0.05). The statistics software used was SPSS 16.0 (SPSS Inc., Chicago, IL, USA). The data are expressed as the mean  ±  SE (*n* = 3), and significant differences are indicated at *p* < 0.05 using different lowercase letters.

## Results

### Toxicities of fluopyram and abamectin against J2

The LC_50_ values of fluopyram and abamectin to the J2 of RKN were 2.53 mg/L and 1.62 mg/L, respectively (Table 1). The LC_50_ values of the different ratio combinations of fluopyram and abamectin were also determined. The 1:5 combination of fluopyram and abamectin had the lowest LC_50_ value (0.64 mg/L), and the CTC was 268. This synergistic effect was the best among the different combinations. Thus, the combination of active ingredients at the 1:5 ratio of fluopyram (41.7% SC) and abamectin (1.8% EC) was selected for the pot and field trials.

**Table 1.** Toxicities of fluopyram and abamectin against second-stage juveniles (J2) of *Meloidogyne incognita* (48 h).

Pesticides	Mass ratio of active ingredient	Slope ± SE	LC_50_ (mg/L)	95% Confidence limits (mg/L)	*r*	*p*	*χ*^2^	CTC
Fluopyram	–	1.43 ± 0.22	2.53	1.41–4.52	0.96	0.0082	120.28	–
Abamectin	–	1.56 ± 0.14	1.62	1.34–1.95	0.98	0.0017	49.69	–
Fluopyram:Abamectin	1:1	1.65 ± 0.26	0.99	0.71–1.40	0.96	0.0084	139.20	198
	1:3	2.04 ± 0.40	0.78	0.50–1.24	0.95	0.0150	92.22	227
	1:5	1.29 ± 0.29	0.64	0.37–1.12	0.93	0.0212	321.94	268
	3:1	1.89 ± 0.18	1.99	1.64–2.42	0.98	0.0021	79.68	111
	5:1	1.81 ± 0.22	3.16	2.42–4.11	0.98	0.0038	100.36	73

Note: CTC was co-toxicity coefficient.

### Pot trials

#### Growth rate of indicators in different treatments

The pot trial results showed that the plant height, stem diameter, root fresh weight, shoot fresh weight, and root length of the tomatoes in the different treatments were all increased compared to the Ctrl at 60 DAT. The combination of fluopyram (41.7% SC) and abamectin (1.8% EC) at 450 g a.i./ha (FA3) had the best protective effect among all the treated groups. The effect of the combination of fluopyram (41.7% SC) and abamectin (1.8% EC) at 337.5 g a.i./ha (FA2) was similar to that of fluopyram (41.7% SC) at 450 g a.i./ha (F) and abamectin (1.8% EC) at 375 g a.i./ha (A). However, the effect of the combination of fluopyram (41.7% SC) and abamectin (1.8% EC) at 225 g a.i./ha (FA1) was lower than that of fluopyram and abamectin used alone (Table 2).

**Table 2.** Effects of the different treatments on the physiological growth indicators of tomato in pot trials (60 DAT).

	Growth rate (%)				
Code	Plant height	Stem diameter	Fresh root weight	Fresh shoot weight	Root length
F	17.44 ± 5.23 ab	9.84 ± 3.24 ab	28.71 ± 9.09 ab	29.51 ± 10.29 a	15.51 ± 6.38 a
A	10.62 ± 6.26 ab	3.48 ± 9.68 b	22.30 ± 4.90 b	14.34 ± 8.82 abc	11.23 ± 1.91 a
FA1	8.66 ± 4.35 b	2.50 ± 6.63 b	7.93 ± 1.77 c	8.83 ± 8.39 c	5.62 ± 5.11 a
FA2	13.51 ± 7.34 ab	6.71 ± 3.36 ab	27.57 ± 9.01 ab	24.14 ± 13.95 ab	15.51 ± 1.59 a
FA3	20.84 ± 4.71 a	14.89 ± 6.73 a	40.30 ± 4.34 a	29.78 ± 2.56 a	21.57 ± 8.34 a
Ctrl	–	–	–	–	–

Note: DAT was days after transplanting. Capital letter F was fluopyram (41.7% SC) at 450 g a.i./ha; A was abamectin (1.8% EC) at 375 g a.i./ha; FA1, FA2 and FA3 were the combination of fluopyram (41.7% SC) and abamectin (1.8% EC) at 225, 337.5 and 450 g a.i./ha, respectively. Also, Ctrl was untreated control. Data are expressed as the mean ± SE. Values followed by different lowercase letters are significantly different (*p* < 0.05).

#### Soil nematode control efficacy in different treatments

**Figure 2:**
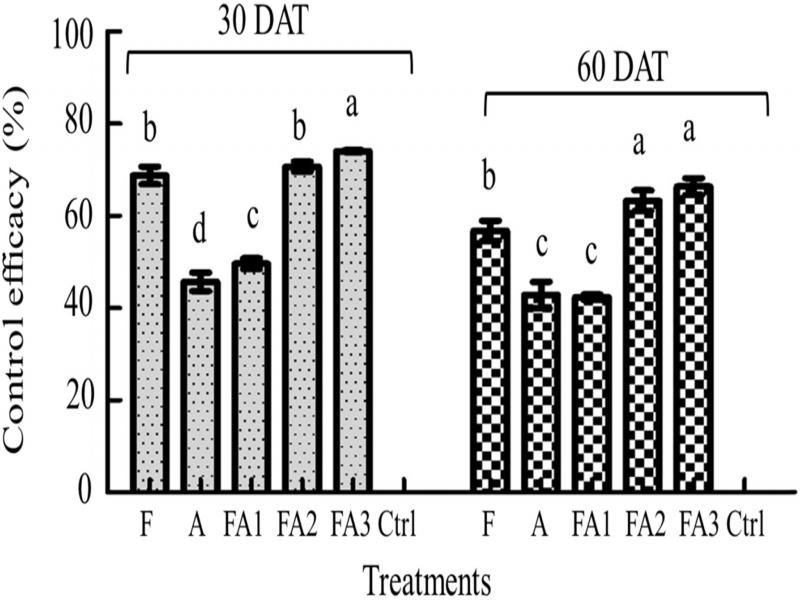
Control efficacy of the different treatments based on the number of *M. incognita* in pot trials. DAT was days after transplanting. Data are expressed as the mean ± SE. Different lowercase letters indicate significant differences (*p* < 0.05). The abbreviations of the capital letters were the same as those in Table 2.

The pot trial results showed that the control efficacy of the FA3 combination was 74.06% at 30 DAT, which was the best effect among all treatments. The control efficacy of the FA2 combination was similar to that of treatment group F, which was higher than that of treatment groups FA1 and A at 30 DAT. At 60 DAT, the control efficacy of the FA3 or FA2 combinations was higher than the efficacies of the other treatment groups and significantly different from those of the F, A, and FA1 treatments (Figure 2).

#### RGI control efficacy in different treatments

**Figure 3:**
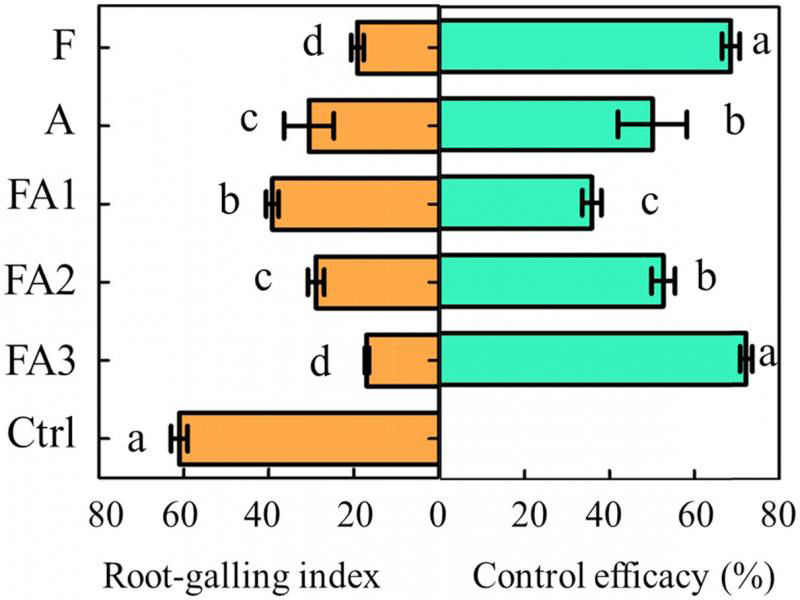
Control efficacy of the different treatments based on the root-galling index in pot trials (60 DAT). Data are expressed as the mean ± SE. Different lowercase letters indicate significant differences (*p* < 0.05). The abbreviations of the capital letters were the same as those in Table 2.

The pot trial results showed that the RGI in treatments FA3 and F decreased significantly compared to that in the Ctrl. The control efficacy of treatment FA3 was 71.98% at 60 DAT, which was similar to that of treatment group F and significantly higher than that of treatments A, FA1, and FA2. The control efficacy of treatment FA2 was similar to that of treatment A and significantly higher (*p*  <  0.05) than that of treatment FA1. The control efficacy of treatment FA1 was the lowest among the five treatments (Figure 3).

#### Root activity in the different treatments

**Figure 4:**
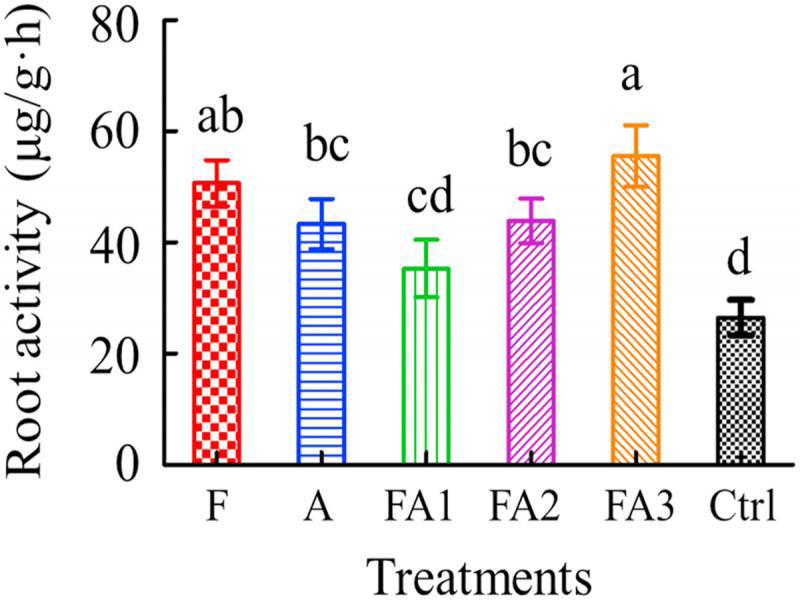
Root activity of tomato in the different treatments in pot trials. Data are expressed as the mean ± SE. Different lowercase letters indicate significant differences (*p* < 0.05). The abbreviations of the capital letters were the same as those in Table 2.

The root activities of tomato increased in the pesticide treatments at 60 DAT. The root activity of treatment FA3 was 2.09-fold higher than that of the Ctrl, and significantly higher (*p*  <  0.05) than that of treatments A, FA1, and FA2. The root activities of treatments FA1 and FA2 were significantly higher than that of the Ctrl (Figure 4).

### Field trials

#### Soil nematode control efficacy in different field treatments

The results of field trials at LW (2018) showed that the number of nematodes decreased in the five treatments at 30 DAT. The control efficacy of treated group FA3 was 53.04%, which was significantly higher than that of other treated groups. The control efficacy of all treatment groups at 60 DAT was better than that at 30 DAT. The control efficacy of treatments FA3 and FA2 were 53.19 and 58.13%, respectively, and were significantly higher than those of the other treatments (Table 3). The results of the field trials at DY (2019) were similar to those of LW (2018). However, the initial average number of southern RKN in the field soil of DY (2019) was lower than that in LW (2018). There was no significant difference between all the treatments, although the control efficacy was slightly higher than those of most treatments (Table 3).

**Table 3.** Control efficacy of the different treatments based on the number of *Meloidogyne incognita* in field soil.

			Control efficacy (%)	
Site/year	Code	Initial no. of nematodes (nematodes/100 g soil)	30 DAT	60 DAT
LW/2018	F	1780.88 ± 203.11 a	29.52 ± 1.60 b	37.31 ± 7.09 b
	A	1439.19 ± 201.26 a	15.43 ± 3.76 c	29.77 ± 1.73 b
	FA1	1520.80 ± 573.47 a	12.86 ± 3.04 c	17.75 ± 4.75 c
	FA2	1441.61 ± 124.94 a	35.12 ± 1.89 b	53.19 ± 1.75 a
	FA3	1658.66 ± 277.33 a	53.04 ± 5.27 a	58.13 ± 3.75 a
	Ctrl	1638.89 ± 411.11 a	–	–
DY/2019	F	1288.33 ± 223.45 a	31.50 ± 1.80 b	38.67 ± 3.79 b
	A	1457.41 ± 159.75 a	14.67 ± 2.51 c	30.67 ± 2.52 b
	FA1	1523.16 ± 188.47 a	15.70 ± 2.25 c	22.46 ± 3.50 c
	FA2	1287.33 ± 182.67 a	38.33 ± 2.30 b	52.53 ± 4.10 a
	FA3	1461.12 ± 160.25 a	57.50 ± 3.61 a	59.73 ± 1.60 a
	Ctrl	1314.16 ± 110.32 a	–	–

Note: LW and DY were the area of Laiwu in Jinan City and Daiyue in Tai’an City, Shandong Province, China. The abbreviations of other capital letters were the same as those in Table 2. Data are expressed as the mean ± SE. Values followed by different lowercase letters are significantly different (*p*  <  0.05).

#### RGI control efficacy and yield in different field treatments

**Figure 5:**
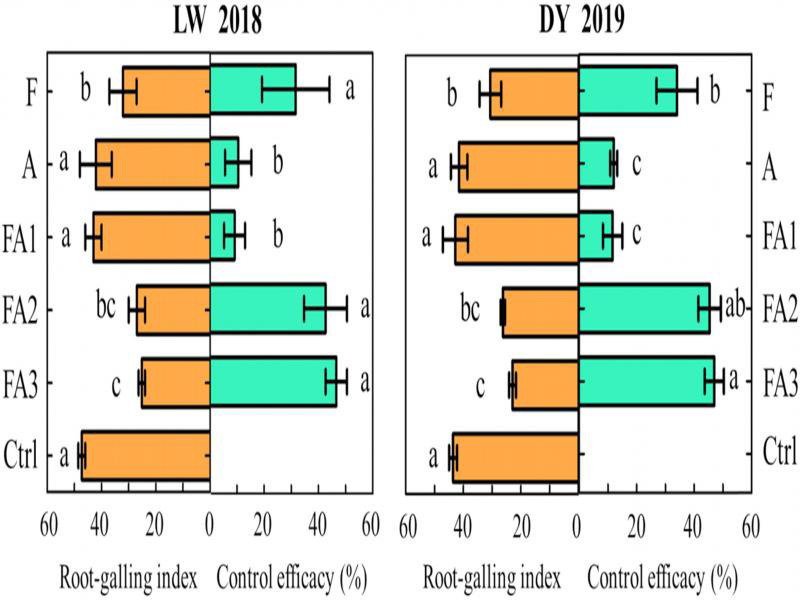
Control efficacy of the different treatments on the root-galling index in field trials (at the end of tomato production, LW 2018 and DY 2019). LW and DY were the area of Laiwu in Jinan City and Daiyue in Tai’an City, Shandong Province, China. Data are expressed as the mean ± SE. Different lowercase letters indicate significant differences (*p* < 0.05). The abbreviations of the capital letters were the same as those in Table 3.

At the end of the tomato production season, the field trials in LW (2018) showed that the RGI in treatments FA3, FA2, and F decreased significantly (*p*  <  0.05) compared to the Ctrl. The control efficacies of treatments FA3 and FA2 were 46.64% and 42.69%, respectively, and these were significantly higher than those of treatments FA1 and A. The control efficacies of treatments FA1 and A were the lowest among the five treatments (Figure 5). The results of the DY (2019) field trial were similar to those of LW (2018); however, there was a significant difference between treatments FA3 and F in DY. This indicated that when using the same dosage, the control efficacy of the fluopyram (41.7% SC) and abamectin (1.8% EC) combination was better than that of fluopyram (41.7% SC) alone (Figure 5).

**Figure 6:**
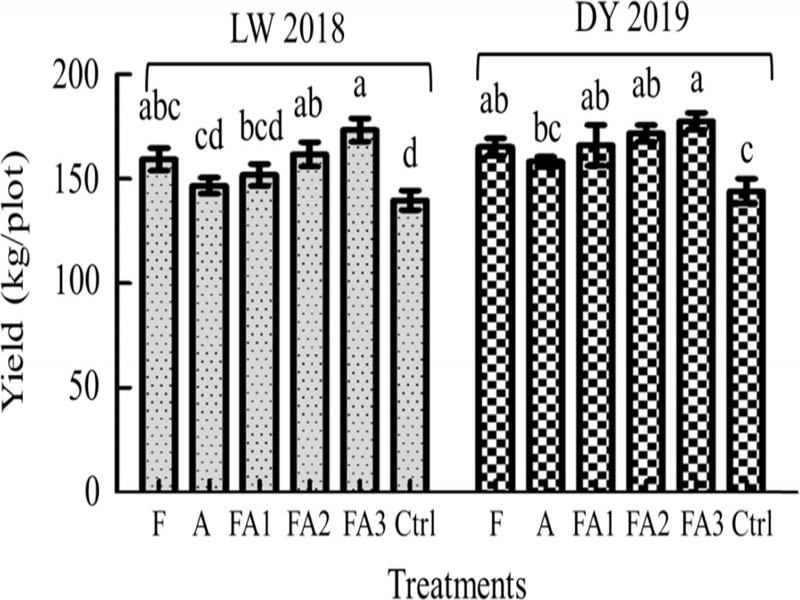
Yield of the different treatments in field trials (LW 2018 and DY 2019). LW and DY were the area of Laiwu in Jinan City and Daiyue in Tai’an City, Shandong Province, China. Data are expressed as the mean ± SE. Different lowercase letters indicate significant differences (*p* < 0.05). The abbreviations of the capital letters were the same as those in Table 3.

There were significant increases in tomato yields between the treatments FA3, FA2, and F, and the Ctrl. The yields of treatments FA3 and FA2 were higher by 24.07 and 15.74%, respectively, compared to the Ctrl group in LW (2018). The yields of FA3 and FA2 were higher by 23.22 and 19.18%, respectively, compared to the Ctrl in DY (2019) (Figure 6).

## Discussion

The southern root-knot nematode *M. incognita* is widely distributed in solar greenhouse tomato production in northern China. Typical root symptoms are observed in the absence of nematicides or as a result of control failures (Lu et al., 2017). This has a serious impact on tomato rotation cultivation. Currently, there are a few of resistant tomatoes with the Mi gene; however, the use of resistant tomato cultivars or resistant rootstocks are an un-effective control option for some RKN populations, and they are commercially unavailable in northern China, and, non-chemical control measures are difficult and often unsatisfactory (Liu et al., 2020). Therefore, the use of environmentally friendly chemical nematicides alone or in combination with biogenic natural product pesticides is a possible way to reduce nematode population densities. This would allow better plant development under infested field conditions (Arita et al., 2020). The combinations of pesticides are commonly used and they have become an important part of integrated pest management.

We determined that the LC_50_ values of fluopyram and abamectin to the J2 of RKN were 2.53 and 1.62 mg/L, respectively. These results are similar to previously reported findings (Ji et al., 2019; Li et al., 2018). To control *M. incognita* more efficiently, we combined fluopyram with abamectin and found that fluopyram and abamectin at a ratio of 1:5 had the highest toxicity and the best synergistic effect among the tested combinations. Three different dosages of the combination (fluopyram 41.7% SC and abamectin 1.8% EC) were used to study their control of soil nematodes and RGI in pot and field trials. Regardless of whether fluopyram or abamectin was used alone or combined at a mass ratio of 1:5, all pesticide treatments increased the height, stem diameter, root fresh weight, shoot fresh weight, and root length of tomato plants. The combination of fluopyram and abamectin produced a significant dose-response on treated nematodes. Fluopyram can effectively reduce the number of root-knots, increase plant height and stem diameter, and promote the growth of tomatoes in greenhouses (Li et al., 2020).

The application of fluopyram alone or combined with other pesticides as a nematicide for the control of nematodes has been widely studied (Faske and Hurd, 2015; Watson et al., 2020; Dahlin et al., 2019; Roth et al., 2020; Feist et al., 2020). The control effects of abamectin on nematodes have also been reported. Abamectin can increase shoot weight, reduce galling, and decrease the reproduction of *M. incognita* in tomato (Lopez-Perez et al., 2011). When exposed to sublethal concentrations of abamectin, the infectivity of *M. incognita* and *Rotylenchulus reniformis* Linford on tomato was reduced (Faske and Starr, 2006). Abamectin combined with azoxystrobin had a good control effect on *Trichodorus obtusus* Cobb in Zoysia grass (Shaver et al., 2016). Abamectin combined with 1,3-dichloropropene (1,3-D) effectively inhibited *M. incognita*. The control efficacy of the combined treatment was higher than that of 1,3-D used alone and the tomato yields were increased (Qiao et al., 2014).

We found that abamectin had higher toxicity than fluopyram to the J2 of RKN. The best synergistic combination of the two actives was fluopyram: abamectin at a 1:5 ratio. However, the control effect of abamectin (1.8% EC, 375 g a.i./ha) was significantly lower than that of fluopyram (41.7% SC, 450 a.i./ha) in both pot and field trials. One possible reason for this is that the abamectin dose was lower than that of fluopyram. Dosages were established according to the standards registered in China. Another possible reason is that abamectin undergoes photodegradation; has a high affinity to soil particles, low diffusivity, a short half-life in the soil; and has low solubility in water (Halley et al., 1993; Tišler and Eržen, 2006; Dionisio and Rath, 2016). The adsorption, leaching, and mobility of abamectin in the soil can lead to lower effectiveness on nematodes. Also, the 1.8% EC formulation of abamectin may contribute to its low control efficacy in soil (Shaver et al., 2016; Li et al., 2018). When fluopyram was combined with abamectin in the trials, we found that the RGI decreased, and the control effect and tomato yield significantly increased. We believe that this is because fluopyram promotes abamectin diffusion during the process of transport and uptake from the soil and roots, and abamectin has high toxicity to RKN. However, the mechanism requires further clarification.

### Ethical approval

This study did not involve any situations using human participants or animals.

### Author contributions

C-B X conceived and designed research. Q-Q L, J-J L, Q-T Y, and Z-Y S conducted experiments and analyzed data. All authors contributed with the discussion of the results. C-B X, Q-Q L, and J-J L wrote the manuscript. All authors read and approved this manuscript.

## References

[ref001] Abad, P. , Gouzy, J. , Aury, J. M. , Castagnone-Sereno, P. , Danchin, E. G. J. , Deleury, E ., et al., 2008. Genome sequence of the metazoan plant-parasitic nematode *Meloidogyne incognita* . Nature Biotechnology 26:909–915. 10.1038/nbt.1482 18660804

[ref002] Abad-Feuntes, A. , Ceballos-Alcantrallia, E. , Mercader, J. V. , Agulló, C. , Abad-Somovilla, A. and Esteve-Turrillas, F. A. 2015. Determination of succinate dehydrogenase-inhibitor fungicide residues in fruits and vegetables by liquid- chromatography-tandem mass spectrometry. Analytical and Bioanalytical Chemistry. 10.1007/s00216-015-8608-3 25796526

[ref003] 407:4207–4211.

[ref004] Arita, L. Y. , Silva, S. A. and Machado, A. C. Z. 2020. Efficacy of chemical and biological nematicides in the management of *Meloidogyne paranaensis* in *Coffea arabica* . Crop Protection 131:105099.

[ref005] Barker, K. R. , Townshend, J. L. , Bird, G. W. , Thomason, I. J. and Dickson, D. W. 1986. “Determining nematode population responses to control agents”, In Hickey, K. D. (Ed.), Methods for Evaluating Pesticides for Control of Plant Pathogens APS Press, Paul, MN, pp. 283–296.

[ref006] Dahlin, P. , Eder, R. , Consoli, E. , Krauss, J. and Kiewnick, S. 2019. Integrated control of *Meloidogyne incognita* in tomatoes using fluopyram and *Purpureocillium lilacinum* strain 251. Crop Protection 124:104874.

[ref007] Dionisio, A. C. and Rath, S. 2016. Abamectin in soils: analytical methods, kinetics, sorption and dissipation. Chemosphere 151:17–29. 26923238 10.1016/j.chemosphere.2016.02.058

[ref008] Faske, T. R. and Starr, J. L. 2006. Sensitivity of *Meloidogyne incognita* and *Rotylenchulus reniformis* to abamectin. Journal of Nematology 38:240–244. 19259453 PMC2586449

[ref009] Faske, T. R. and Hurd, K. 2015. Sensitivity of *Meloidogyne incognita* and *Rotylenchulus reniformis* to fluopyram. Journal of Nematology 47:316–321. 26941460 PMC4755706

[ref010] Feist, E. , Kearn, J. , Gaihre, Y. , O’Connor, V. and Holden-Dye, L. 2020. The distinct profiles of the inhibitory effects of fluensulfone, abamectin, aldicarb and fluopyram on *Globodera pallida* hatching. Pesticide Biochemistry and Physiology 165:104541. 32359561 10.1016/j.pestbp.2020.02.007

[ref011] Giannakou, I. O. , Karpouzas, D. G. , Anastasiades, I. , Tsiropoulos, N. G. and Georgiadou, A. 2005. Factors affecting the efficacy of non-fumigant nematicides for controlling root- knot nematodes. Pest Management Science 61:961–972. 15983980 10.1002/ps.1081

[ref012] Halley, B. A. , VandenHeuvel, W. J. A. and Wislocki, P. G. 1993. Environmental effects of the usage of avermectins in livestock. Veterinary Parasitology 48:109–125. 8346626 10.1016/0304-4017(93)90149-h

[ref013] Huang, W. K. , Sun, J. H. , Cui, J. K. , Wang, G. F. , Kong, L. A. , Peng, H. , Chen, S. L. and Peng, D. L. 2014. Efficacy evaluation of fungus *Syncephalastrum racemosum* and nematicide avermectin against the rootknot nematode *Meloidogyne incognita* on cucumber. PLoS ONE 9:e89717. 24586982 10.1371/journal.pone.0089717 PMC3933638

[ref014] Huang, W. K. , Wu, Q. S. , Peng, H. , Kong, L. A. , Liu, S. M. , Yin, H. Q. , Cui, R. Q. , Zhan, L. P. , Cui, J. K. and Peng, D. L. 2016. Mutations in Acetylcholinesterase2 (ace2) increase the insensitivity of acetylcholinesterase to fosthiazate in the root-knot nematode *Meloidogyne incognita* . Scientific Reports 6:38102. 27897265 10.1038/srep38102 PMC5126670

[ref016] Huang, X. P. , Luo, J. , Li., B. X. , Song, Y. F. , Mu, W. and Liu, F. 2019b. Bioactivity, physiological characteristics and efficacy of the SDHI fungicide pydiflumetofen against *Sclerotinia sclerotiorum* . Pesticide Biochemistry and Physiology 160:70–78. 31519259 10.1016/j.pestbp.2019.06.017

[ref015] Huang, B. , Wang, Q. , Guo, M. X. , Fang, W. S. , Wang, X. N. , Wang, Q. X. , Yan, D. D. , OuYang, C. B. , Li, Y. and Cao, A. C. 2019a. The synergistic advantage of combining chloropicrin or dazomet with fosthiazate nematicide to control root-knot nematode in cucumber production. Journal of Integrative Agriculture 18:2093–2106.

[ref017] Hussey, R. S. and Barker, K. R. 1973. A comparison of methods of collecting inocula of *Meloidogyne* spp., including a new technique. Plant Disease Report 57:1025–1028.

[ref018] ICAMA 2019. Electronic manual of insecticides, available at: http://www.icama.org.cn/hysj/index.jhtml (accessed August 26, 2019).

[ref019] Ji, X. X. , Li, J. J. , Dong, B. , Zhang, H. , Zhang, S. A. and Qiao, K. 2019. Evaluation of fluopyram for southern root-knot nematode management in tomato production in China. Crop Protection 122:84–89.

[ref020] Jones, J. G. , Kleczewski, N. M. , Desaeger, J. , Meyer, S. L. F. and Johnson, G. C. 2017. Evaluation of nematicides for southern root-knot nematode management in lima bean. Crop Protection 96:151–157.

[ref022] Li, B. X. , Ren, Y. P. , Zhang, D. X. , Xu, S. Y. , Mu, W. and Liu, F. 2018. Modifying the formulation of abamectin to promote its efficacy on southern root-knot nematode (*Meloidogyne incognita*) under blending-of-soil and root-irrigation conditions. Journal of Agriculture and Food Chemistry 66:799–805. 10.1021/acs.jafc.7b04146 29240417

[ref021] Li, H. S. 2000. Principles and techniques of plant physiological and biochemical experiments 2nd ed., Higher Education Press, Beijing, pp. 122–124.

[ref023] Li, J. J. , Meng, Z. , Li, N. , Dong, B. , Ji, X. X. , Zhang, S. A. and Qiao, K. 2020. Evaluating a new non-fumigant nematicide fluopimomide for management of southern root-knot nematodes in tomato. Crop Protection 129:105040.

[ref024] Liu, G. Y. , Lin, X. , Xu, S. Y. , Liu, G. , Liu, F. and Mu, W. 2020. Screening, identification and application of soil bacteria with nematicidal activity against root-knot nematode (*Meloidogyne incognita*) on tomato. Pest Management Science 76:2217–2224. 31970922 10.1002/ps.5759

[ref025] Lopez-Perez, J. A. , Edwards, S. and Ploeg, A. 2011. Control of root-knot nematodes on tomato in stone wool substrate with biological nematicides. Journal of Nematology 43:110–117. 22791920 PMC3380465

[ref026] Lu, H. B. , Xu, S. Y. , Zhang, W. J. , Xu, C. M. , Li, B. X. , Zhang, D. X. , Mu, W. and Liu, F. 2017. Nematicidal activity of trans-2-hexenal against southern root-knot nematode (*Meloidogyne incognita*) on tomato plants. Journal of Agriculture and Food Chemistry 65:544–550. 10.1021/acs.jafc.6b04091 28048941

[ref027] Mao, X. F. , Li, H. X. , Chen, X. Y. and Hu, F. 2004. Extraction efficiency of soil nematodes by different methods. Chinese Journal of Ecology 23:149–151, (in Chinese).

[ref028] Nicol, J. M. , Turner, S. J. , Coyne, D. L. , Nijs, L. , Hockland, S. and Maafi, Z. T. 2011. “Current nematode threats to world agriculture”, Genomics and molecular genetics of plant-nematode interactions Springer, Dordrecht and South Holland, pp. 21–43.

[ref029] Pinkerton, J. and Finn, C. E. 2005. Responses of strawberry species and cultivars to the rootlesion and northern root-knot nematodes. Hortscience 40:33–38.

[ref030] Putter, I. , Maconnel, J. G. , Preiser, F. A. , Haidri, A. A. , Ristich, S. S. and Dybas, R. A. 1981. Avermectins: novel insecticides, acaricides and nematicides from a soil microorganism. Experientia 37:963–964.

[ref031] Qiao, K. , Zhang, H. , Wang, H. , Ji, X. and Wang, K. 2011. Efficacy of aluminium phosphide as a soil fumigant against nematode and weed in tomato crop. Scientia Horticulturae 130:570–574.

[ref032] Qiao, K. , Liu, X. , Wang, H. Y. , Xia, X. M. , Ji, X. X. and Wang, K. Y. 2012. Effect of abamectin on root-knot nematodes and tomato yield. Pest Management Science 68:853–857. 22395950 10.1002/ps.2338

[ref033] Qiao, K. , Duan, H. M. , Wang, H. Y. , Wang, Y. , Wang, K. Y. and Wei, M. 2014. The efficacy of the reduced rates of 1,3-D + abamectin for control of *Meloidogyne incognita* in tomato production in China. Scientia. Horticulturae 178:248–252.

[ref034] Roth, M. G. , Jacobs, J. L. , Napieralski, S. , Byrne, A. M. , Stouffer-Hopkins, A. , Warner, F. and Chilvers, M. I. 2020. Fluopyram suppresses population densities of *Heterodera glycines* in field and greenhouse studies in Michigan. Plant Disease 104:1305–1311. 32155114 10.1094/PDIS-04-19-0874-RE

[ref035] Shaver, B. R. , Agudelo, P. and Martin, S. B. 2016. Use of abamectin and azoxystrobin for managing stubby-Root nematode (*Trichodorus obtusus* Cobb) damage to zoysiagrass. Crop Science 56:1330–1336.

[ref036] Shi, X. G. , Qiao, K. , Li, B. T. and Zhang, S. A. 2019. Integrated management of *Meloidogyne incognita* and *Fusarium oxysporum* in cucumber by combined application of abamectin and fludioxonil. Crop Protection 126:104922.

[ref038] Sun, M. H. , Gao, L. , Shi, Y. X. , Li, B. J. and Liu, X. Z. 2006. Fungi and actinomycetes associated with *Meloidogyne* spp. eggs and females in China and their biocontrol potential. Journal of Invertebrate Pathology 93:22–28. 16737708 10.1016/j.jip.2006.03.006

[ref037] Sun, Y. P. and Johnson, E. R. 1960. Analysis of joint action of insecticides against house flies. Journal of Economic Entomology 53:887–892.

[ref039] Tian, B. Y. , Cao, Y. and Zhang, K. Q. 2015. Metagenomic insights into communities, functions of endophytes and their associates with infection by root-knot nematode, *Meloidogyne incognita*, in tomato roots. Scientific Reports 5:17087. 26603211 10.1038/srep17087 PMC4658523

[ref040] Tišler, T. and Eržen, N. K. 2006. Abamectin in the aquatic environment. Ecotoxicology 15:495–502. 16741677 10.1007/s10646-006-0085-1

[ref041] Watson, T. T. and Desaeger, J. A. 2019. Evaluation of non-fumigant chemical and biological nematicides for strawberry production in Florida. Crop Protection 117:100–107.

[ref042] Watson, T. T. , Noling, J. W. and Desaeger, J. A. 2020. Fluopyram as a rescue nematicide for managing sting nematode (*Belonolaimus longicaudatus*) on commercial strawberry in Florida. Crop Protection 132:105–108.

[ref043] Zhang, D. L. , Wang, H. Y. , Ji, X. X. , Wang, K. Y. , Wang, D. and Qiao, K. 2017. Effect of abamectin on the cereal cyst nematode (CCN, *Heterodera avenae*) and wheat yield. Plant Disease 101:973–976. 30682934 10.1094/PDIS-10-16-1441-RE

